# Time Course and Clinical Relevance of Neurological Deterioration After Endovascular Recanalization Therapy for Anterior Circulation Large Vessel Occlusion Stroke

**DOI:** 10.3389/fnagi.2021.651614

**Published:** 2021-06-29

**Authors:** Zibao Li, Hongchuan Zhang, Jian Han, Zhaohu Chu, Shoucai Zhao, Qian Yang, Xianjun Huang, Zhiming Zhou

**Affiliations:** ^1^Department of Neurology, Yijishan Hospital of Wannan Medical College, Wuhu, China; ^2^Department of Radiology, Yijishan Hospital of Wannan Medical College, Wuhu, China; ^3^Department of Neurology, Huangshan City People’s Hospital, Huangshan, China

**Keywords:** neurological deterioration, time course, stroke, recanalization, odds ratio

## Abstract

Neurological deterioration (ND) is a devastating complication for patients with ischemic stroke after endovascular recanalization therapy (EVT). We aimed to investigate the time course and clinical relevance of ND after EVT. Consecutive patients with acute ischemic stroke who underwent EVT for large arterial occlusions of the anterior cerebral circulation were enrolled. The National Institutes of Health Stroke Scale (NIHSS) scores were assessed before EVT, at the end of EVT, at 24 h (d1), on day 3 (d3), on day 15 (d15), at discharge and anytime when ND was indicated. ND was defined as an increase of ≥ 4 points in the NIHSS score and was divided into acute ND (AD, within 24 h), subacute ND (SD, d1–d3), and delayed ND (DD, d3–d15 or discharge). Using multivariable logistic regression analysis, we explored predictors and outcomes of ND at different time periods. As a result, of 343 patients, 129 (37.6%) experienced ND, including 90 (26.2%) with AD, 27 (7.9%) with SD and 12 (3.5%) with DD. Multivariable logistic regression analysis revealed that history of hypertension, cardioembolic stroke, lower Alberta Stroke Program Early Computed Tomography Score (ASPECTS), and poor collaterals were significantly associated with an increased risk of AD; history of hypertension, lower ASPECTS, poor collaterals, and unsuccessful recanalization, with SD; and high admission NIHSS score, with DD. In addition, patients who experienced AD (OR = 10.22, *P* < 0.001), SD (OR = 15.89, *P* = 0.004), or DD (OR = 8.31, *P* = 0.015) were more likely to have poor outcomes. ND was a strong predictor of poor stroke outcomes. Management of related risk factors at different ND time periods might improve the prognosis of EVT.

## Introduction

With the aging of global population, stroke has become the second leading cause of disability-adjusted life-years (DALYs) for older adults underlying the need to deal with disabling outcome (GBD 2019 Diseases and Injuries Collaborators, 2020). Recently, several randomized controlled trials have confirmed the safety and efficacy of endovascular recanalization therapy (EVT) for patients with large arterial occlusion strokes in anterior cerebral circulation (Berkhemer et al., 2015; Campbell et al., 2015; Goyal et al., 2015; Jovin et al., 2015; Saver et al., 2015). However, individual responses to EVT vary widely and clinical evolution is largely unpredictable. Less than half of patients achieve functional independence, while others become dependent or die at 90 days (Goyal et al., 2016). Previous studies had already suggested that early neurological deterioration (ND) predicted poor functional outcomes after EVT (Zhang et al., 2018; Kim et al., 2019a). The perioperative management of patients with EVT is a continuous and refined process during hospital. The incidence, predictors, and outcomes of ND in different time periods might be different. Thus, study of ND in different time periods after EVT contributed to a better individualized management.

Thus far, only few studies have reported the characteristics of ND after EVT. Regardless of the different definitions used, Kim et al. (2019a) showed that early ND occurs in 35.2% of patients and is significantly associated with large artery atherosclerosis (LAA) stroke, unsuccessful recanalization and a high National Institutes of Health Stroke Scale (NIHSS) score after EVT. Zhang et al. (2018) found that early ND occurred in 40.2% of patients, and high admission systolic blood pressure (SBP) and unsatisfactory recanalization of occluded arteries contributed to early ND. However, the clinical relevance for different ND time periods during hospitalization was not systematically investigated in either study. Therefore, we performed this prospective observational study to characterize the incidence, predictors, and outcomes of ND during different time periods to achieve refined management after EVT during hospitalization and to enrich enrollment in clinical trials of research interventions to decrease progression.

## Materials and Methods

### Study Population

This study was a retrospective analysis of a prospectively collected stroke database. Consecutive patients with acute ischemic stroke undergoing EVT for large arterial occlusions of the anterior cerebral circulation on computed tomography angiography (CTA), magnetic resonance angiography (MRA), or digital-subtraction angiography (DSA) were enrolled from Yijishan Hospital of Wannan Medical College between May 2015 and September 2020. Patients with a modified Rankin Scale (mRS) score > 2 before the index stroke were excluded from the study. This study was approved by the Ethical Review Board of Yijishan Hospital in Wuhu, China. Written informed consent was obtained from all enrolled patients or their surrogates.

### Endovascular Recanalization Therapy

The protocols of EVT and perioperative management strategies have been described previously (Hao et al., 2017; Huang et al., 2019; Li et al., 2019). Briefly, all patients received local anesthesia. Diazepam or dexmedetomidine was used in some patients who did not cooperate with the operation due to disturbance of consciousness. EVT was performed using a Solitaire stent retriever (Covidien, Irvine, CA, United States) or aspiration thrombectomy (Penumbra system, Alameda, CA, United States) as the first choice. If recanalization of targeting artery was not achieved, stent implantation, balloon dilation, or intra-arterial tirofiban administration were used as remedial measures.

### Clinical and Radiologic Assessment

Good collaterals were defined as >50% filling of the occluded area (Tan et al., 2007). Successful recanalization after EVT was defined as a modified Thrombolysis in Cerebral Infarction (mTICI) score of 2b or 3 (Zaidat et al., 2013). The NIHSS scores were recorded by certified neurologists before EVT, at the end of EVT, at 24 h (d1), on day 3 (d3), on day 15 (d15), at discharge and anytime when ND was indicated. For those patients receiving diazepam or dexmedetomidinem, the time of first postoperative evaluation was delayed to 24 h after EVT. ND was defined as an increase of four points or more in the NIHSS score compared to the best neurological status during hospitalization. We evaluated the time course of ND based on each time node of available clinical evaluations: acute ND (AD, within 24 h), subacute ND (SD, d1–d3), and delayed ND (DD, d3–d15 or discharge).

Non-contrast cranial CT scans were usually performed at 24 and 72 h after EVT or anytime ND was indicated by clinical manifestations. Symptomatic intracranial hemorrhage (sICH) was defined as parenchymal hemorrhage type 2 on non-contrast cranial CT with ND ≥ 4 NIHSS points from baseline (Kim et al., 2019b). A good functional outcome was defined as a mRS score ≤ 2 at 90-day follow-up. All clinical and imaging evaluations were performed in blind by two experienced neurologists. In cases of disagreement, a senior neuroscientist was consulted.

### Statistical Analysis

SPSS software (version 23.0; IBM, Armonk, NY, United States) was used for statistical analysis. Categorical variables are described by frequencies (percentages) and were compared using chi-square or Fisher exact tests. Continuous variables with normal distributions are presented as the mean (standard deviation, SD) and were compared using student t tests. Continuous variables without normal distributions are expressed as the median (interquartile range, IQR) and were compared using Mann-Whitney U tests. The association between potential predictive factors and outcome variables (ND and stroke outcome) was evaluated using logistic regression. Significant (*P* < 0.1) univariate predictive factors were candidates for inclusion in a multivariable logistic regression. A two-sided *P* < 0.05 was considered statistically significant.

## Results

### Baseline Characteristics

We enrolled 343 patients with EVT in our study (Table 1). ND occurred in 129 (37.6%) patients, including 90 (26.2%) with AD, 27 (7.9%) with SD and 12 (3.5%) with DD, while 214 (62.4%) patients had no ND. Three patients without CT scan data in the ND group were discharged due to sudden neurological deterioration. Compared to patients with no ND at any time point during hospitalization, patients with AD were more likely to have an older age (70.7 vs. 67.7 years, *P* = 0.031), higher rate of hypertension (78.9 vs. 65.0%, *P* = 0.016), higher rate of atrial fibrillation (62.2 vs. 43.9%, *P* = 0.004), higher NIHSS score on admission (18 vs. 14, *P* < 0.001), higher rate of an occlusion site in the internal carotid artery (ICA) (57.8 vs. 37.4%, *P* = 0.005), longer procedure time (PT) (79 vs. 60 min, *P* = 0.005), higher rate of cardioembolic stroke (CE) (72.2 vs. 50.9%, *P* = 0.001), lower Alberta Stroke Program Early Computed Tomography Score (ASPECTS) (8 vs. 9, *P* < 0.001), lower rate of good collaterals (23.3 vs. 52.3%, *P* < 0.001) and lower rate of successful reperfusion (61.1 vs. 79.4%, *P* = 0.001). Patients with SD were more likely to have a higher rate of hypertension (88.9 vs. 65.0%, *P* = 0.012), higher rate of diabetes mellitus (29.6 vs. 14.0%, *P* = 0.048), higher NIHSS score on admission (17 vs. 14, *P* = 0.007), lower ASPECTS (7 vs. 9, *P* < 0.001), lower rate of good collaterals (11.1 vs. 52.3%, *P* < 0.001) and lower rate of successful reperfusion (59.3 vs. 79.4%, *P* = 0.027). A higher NIHSS score on admission was significantly associated with DD (17 vs. 14, *P* = 0.034).

**TABLE 1:** Patient characteristics of the subgroups according to neurological deterioration status.

**Variables**	**Any Neurological Deterioration**		***P***	**First Neurological Deterioration vs. No Deterioration by Category**					
	**Yes (*n* = 129)**	**No (*n* = 214)**		**AD (*n* = 90)**	***P***	**SD (*n* = 27)**	***P***	**DD (*n* = 12)**	***P***
**Demographic characteristics**									
Age, years, mean (SD)	69.8 (11.5)	67.7 (10.9)	0.095	70.7 (10.7)	0.031	67.0 (14.1)	0.763	69.5 (10.6)	0.585
Female sex, *n* (%)	60 (46.5)	95 (44.4)	0.702	45 (50.0)	0.371	11 (40.7)	0.719	4 (33.3)	0.452
**Past medical history, *n* (%)**									
Hypertension	104 (80.6)	139 (65.0)	0.002	71 (78.9)	0.016	24 (88.9)	0.012	9 (75.0)	0.551
Diabetes mellitus	27 (20.9)	30 (14.0)	0.096	16 (17.8)	0.404	8 (29.6)	0.048	3 (25.0)	0.390
Atrial fibrillation	78 (60.5)	94 (43.9)	0.003	56 (62.2)	0.004	15 (55.6)	0.253	7 (58.3)	0.329
Antithrombotics	43 (33.3)	53 (24.8)	0.087	31 (34.4)	0.085	8 (29.6)	0.584	4 (33.3)	0.503
**Clinical data**									
Admission SBP, mean (SD)	151 (23)	147 (23)	0.069	151 (23)	0.130	151 (24)	0.322	153 (19)	0.317
Admission DBP, mean (SD)	83 (14)	81 (14)	0.204	83 (15)	0.362	85 (14)	0.219	83 (8)	0.776
Admission NIHSS, median, (IQR)	17 (14−20)	14 (12−18)	<0.001	18 (14−20)	<0.001	17 (14−20)	0.007	17 (14−21)	0.034
IV-rtPA, *n* (%)	16 (12.4)	24 (11.2)	0.740	14 (15.6)	0.296	1 (3.7)	0.327	1 (8.3)	1.000
Occlusion site, *n* (%)			0.021		0.005		0.827		1.000
ICA	68 (52.7)	80 (37.4)		52 (57.8)		12 (44.4)		4 (33.3)	
MCA-M1	52 (40.3)	114 (53.3)		32 (35.6)		13 (48.1)		7 (58.3)	
MCA-M2, ACA	9 (7.0)	20 (9.3)		6 (6.7)		2 (7.4)		1 (8.3)	
TOAST type, *n* (%)			0.003		0.001		0.435		0.695
CE	89 (69.0)	109 (50.9)		65 (72.2)		17 (63.0)		7 (58.3)	
LAA	28 (21.7)	81 (37.9)		14 (15.6)		9 (33.3)		5 (41.7)	
Others	12 (9.3)	24 (11.2)		11 (12.2)		1 (3.7)		0 (0.0)	
**Radiological findings and procedural aspects**									
ASPECTS, median (IQR)	8 (7−9)	9 (8−10)	<0.001	8 (7−9)	<0.001	7 (5−8)	<0.001	9 (8−10)	0.689
OTP, median (IQR)	257(210−300)	270 (222−330)	0.282	245(210−300)	0.114	296(220−360)	0.420	253(200−293)	0.568
PT, median (IQR)	74 (47−105)	60 (44−90)	0.010	79 (46−119)	0.005	80 (54−100)	0.118	51 (46−71)	0.304
Good collaterals, *n* (%)	28 (21.7)	112 (52.3)	<0.001	21 (23.3)	<0.001	3 (11.1)	<0.001	4 (33.3)	0.200
Procedural modes, *n* (%)			0.120		0.146		0.106		0.804
Solitaire FR first	94 (72.9)	152 (71.0)		69 (76.7)		17 (63.0)		8 (66.7)	
Inspiration first	28 (21.7)	37 (17.3)		17 (18.9)		9 (33.3)		2 (16.7)	
Others,	7 (5.4)	25 (11.7)		4 (4.4)		1 (3.7)		2 (16.7)	
Remedial measures, *n* (%)	18 (14.0)	31 (14.5)	0.891	14 (15.6)	0.811	4 (14.8)	0.999	0 (0.0)	0.379
mTICI (2b/3), *n* (%)	80 (62.0)	170 (79.4)	<0.001	55 (61.1)	0.001	16 (59.3)	0.027	9 (75.0)	0.717

*SBP, systolic blood pressure; DBP, diastolic blood pressure; NIHSS, National Institutes of Health Stroke Scale; SD, standard deviation; IQR, Interquartile range; IV-rtPA, intravenous recombinant tissue plasminogen activator; ICA, internal carotid artery; MCA, middle cerebral artery; TOAST, Trial of Org 10172 in acute stroke treatment; CE, cardioembolic; LAA, large artery atherosclerosis; ASPECTS, the Alberta Stroke Program Early Computed Tomography Score; OTP, onset to puncture time; PT, procedural time; mTICI, modified Thrombolysis in Cerebral Infarction.*

### Neurological Deterioration Free Survival Curves

**FIGURE 1:**
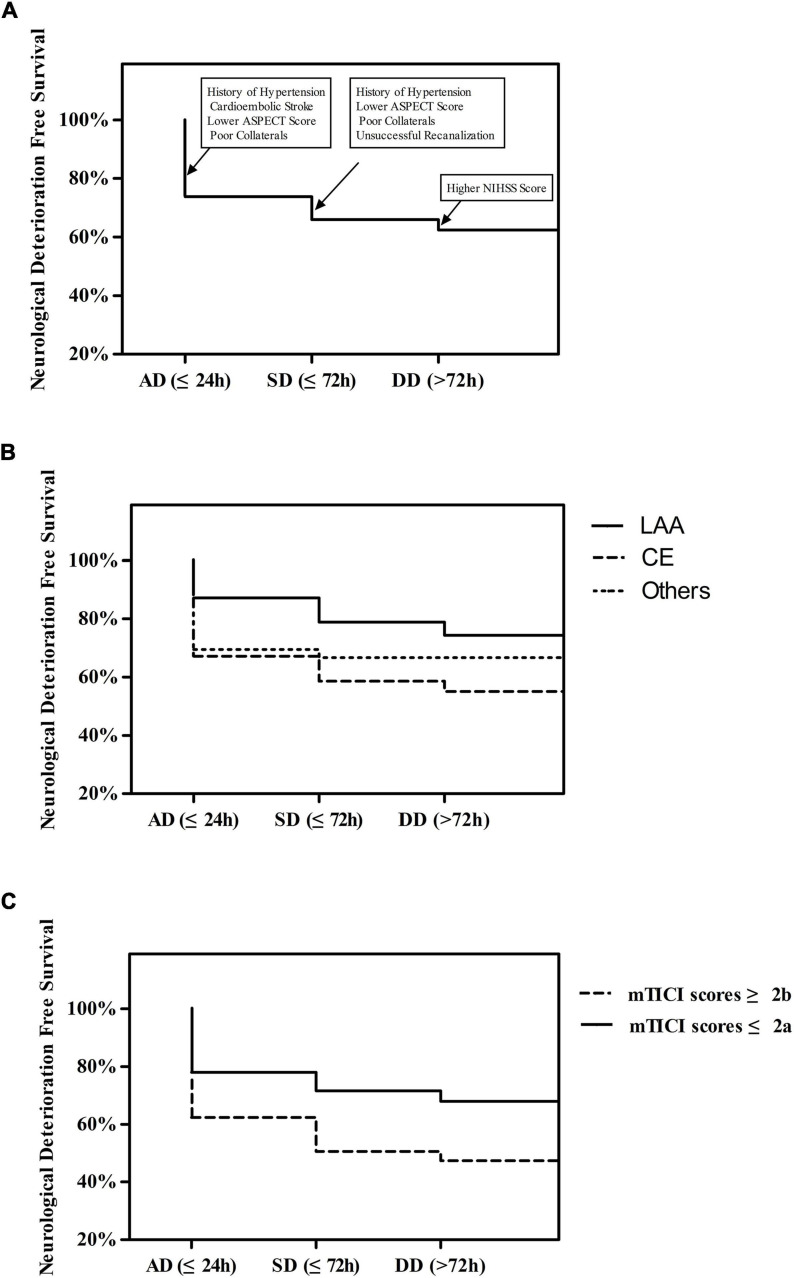
**(A)** Time Course of First Neurological Deterioration. **(B)** The Course of First Neurological Deterioration grouped by TOAST type. **(C)** Time Course of First Neurological Deterioration grouped by mTICI scores.

A Kaplan-Meier curve was performed to test the effects of clinical variables on ND-free survival (Figure 1). ND was most likely to occur within the first 24 h (90/129, 69.8%, Figure 1A). The incidence of ND gradually decreased over time. Compared to patients with LAA stroke grouped by Trial of Org 10172 in Acute Stroke Treatment (TOAST) type, patients with CE stroke and other stroke etiologies had a greater chance of ND (44.9 and 33.3%, respectively, vs. 25.7%, *P* = 0.003, Figure 1B). We also observed that patients with successful recanalization after EVT had a decreased risk of ND (32.0 vs. 52.7%, OR = 0.423, *P* < 0.001, Figure 1C).

### Multivariable Model for Different ND Time Periods

The multivariable analysis of different ND time periods is shown in Table 2 and Figure 1A. AD was significantly associated with history of hypertension (OR = 2.23, 95% CI = 1.09–4.55, *P* = 0.028), TOAST type (LAA vs. CE, OR = 0.38, 95% CI = 0.15–0.97, *P* = 0.043; others vs. CE, OR = 1.34, 95% CI = 0.45–4.02, *P* = 0.602; total *P* = 0.031), high ASPECTS (OR = 0.68, 95%CI = 0.54–0.86, *P* = 0.001), and good collaterals (OR = 0.36, 95% CI = 0.19–0.68, *P* = 0.002). SD was significantly associated with history of hypertension (OR = 6.25, 95% CI = 1.31–29.89, *P* = 0.022), high ASPECTS (OR = 0.47, 95% CI = 0.34–0.66, *P* < 0.001), good collaterals (OR = 0.22, 95% CI = 0.06–0.83, *P* = 0.026), and successful recanalization (OR = 0.26, 95% CI = 0.09–0.78, *P* = 0.016). DD was significantly associated with a high admission NIHSS score (OR = 1.12, 95% CI = 1.00–1.26, *P* = 0.048).

**TABLE 2:** Factors associated with neurological deterioration according to time course.

**Variables**	**Odds ratio**	**Confidence interval**	***P* value**
**AD (≤24 h)**			
Age	1.00	0.97–1.03	0.809
Hypertension	2.23	1.09–4.55	0.028
Atrial fibrillation	0.90	0.40–2.06	0.810
Antithrombotics	1.34	0.70–2.58	0.380
Admission NIHSS	1.02	0.95–1.09	0.633
Occlusion site			0.091
MCA-M1 vs. ICA	0.52	0.28–0.97	0.040
MCA-M2 vs. ICA	0.46	0.15–1.41	0.173
TOAST			0.031
LAA vs. CE	0.38	0.15–0.97	0.043
Others vs. CE	1.34	0.45–4.02	0.602
ASPECTS	0.68	0.54–0.86	0.001
PT	1.01	1.00–1.02	0.071
Good collaterals	0.36	0.19–0.68	0.002
mTICI (2b/3)	0.73	0.36–1.51	0.398
**SD (1–3 days)**			
Hypertension	6.25	1.31–29.89	0.022
Diabetes mellitus	2.70	0.86–8.41	0.088
Admission NIHSS	0.97	0.86–1.08	0.567
ASPECTS	0.47	0.34–0.66	<0.001
Good collaterals	0.22	0.06–0.83	0.026
mTICI (2b/3)	0.26	0.09–0.78	0.016
**DD (>3 days)**			
Admission NIHSS	1.12	1.00–1.26	0.048

*AD, acute neurological deterioration; SD, subacute neurological deterioration; DD, delayed neurological deterioration; NIHSS, National Institutes of Health Stroke Scale; ICA, internal carotid artery; MCA, middle cerebral artery; PT, procedural time TOAST, Trial of Org 10172 in acute stroke treatment; CE, cardioembolic; LAA, large artery atherosclerosis; ASPECTS, the Alberta Stroke Program Early Computed Tomography Score; mTICI, modified thrombolysis in cerebral infarction.*

### Association Between the ND Time Course and Stroke Outcomes

**FIGURE 2:**
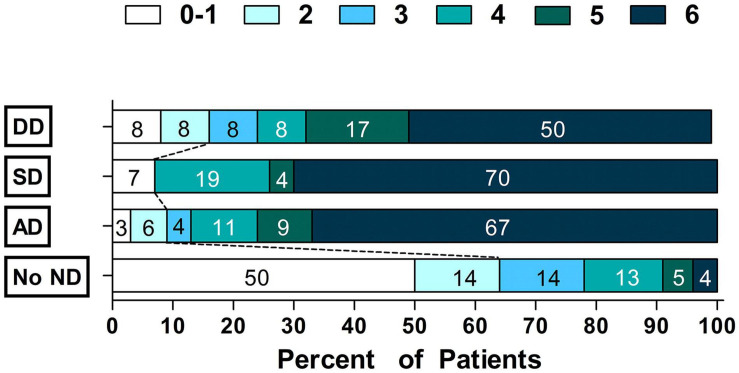
Scores on the modified Rankin Scale grouped by Time Courseof Neurological Deterioration status.

Compared to patients with no ND, patients with ND were more likely to have poor outcomes (90.7 vs. 36.4%, *P* < 0.001). After adjustment for potential confounders (Supplementary Table 1), AD (OR = 10.22, 95% CI = 4.07–25.68, *P* < 0.001), SD (OR = 15.89, 95% CI = 2.47–102.14, *P* = 0.004) and DD (OR = 8.31, 95% CI = 1.51–45.90, *P* = 0.015) were significantly associated with poor outcomes (Table 3 and Figure 2).

**TABLE 3:** Association between time course of ND and poor outcome.

**Time course of ND**	**mRS > 2**		
	**Odds ratio**	**Confidence interval**	***P* value**
**ND vs. no ND**			
AD	10.22	4.07–25.68	<0.001
SD	15.89	2.47–102.14	0.004
DD	8.31	1.51–45.90	0.015

*ND, neurological deterioration; AD, acute neurological deterioration; SD, subacute neurological deterioration; DD, delayed neurological deterioration; mRS, modified Rankin Scale. Adjusted for age, sex, diabetes mellitus, atrial fibrillation, antithrombotics, admission systolic blood pressure (SBP), National Institutes of Health Stroke Scale (NIHSS) score, occlusion site, Trial of Org 10172 in acute stroke treatment (TOAST), the Alberta Stroke Program Early Computed Tomography Score (ASPECTS), procedural time (PT), collateral score, procedural modes, modified Thrombolysis in Cerebral Infarction (mTICI).*

## Discussion

Our study demonstrated that 37.6% of stroke patients experienced ND after EVT during hospitalization, especially in the first 24 h. We also revealed a striking association between ND at different time periods (AD, SD, and DD) and poor prognosis. Clinical strategies focused on prevention of different ND time courses are a logical step to improve outcomes after EVT.

Previous studies indicated that early ND occurs in 35.2–40.2% of patients, most of which occurs within 72 h. This was similar to the incidence in our study despite the different definitions (Zhang et al., 2018; Kim et al., 2019a). Kim et al. (2019a) found that more than half of patients with ICA occlusion experienced ND, which was attributed to the mechanisms of symptomatic hemorrhage, ischemia progression, and brain edema. The risk factors for ND were LAA stroke for ischemia progression, and successful recanalization and NIHSS score after thrombectomy for hemorrhage or brain edema. These findings are generally in line with our results. In this study, 52.7% of patients with ICA occlusion experienced ND, indicating that they were especially susceptible to secondary neuronal injury after EVT. Successful recanalization was also a protective factor for SD, which was consistent with the results from a clinical study in China (Zhang et al., 2018). We found that a high NIHSS score on admission was the only risk factor for DD, suggesting that the effects of baseline risk factors and procedure-related factors on ND elapse over time after EVT. However, compared to CE stroke, LAA stroke was a protective factor for AD, which was inconsistent with the results of Kim et al. (2019a). We speculated that this difference may be due to different ethnic groups, different definitions of ND, and different classification methods of ND. We found that CE stroke patients in our study had poor collaterals (CE vs. LAA, 69.9 vs. 30.1%, respectively, *P* = 0.015), while poor collaterals was a risk factor for both AD and SD. Previous studies indicated that poor collaterals was associated with a lower recanalization rate (Bang et al., 2011; Liebeskind et al., 2014a,b; Leng et al., 2016b), ischemia progression (Campbell et al., 2013; Chen et al., 2019), a higher rate of sICH (Liebeskind et al., 2014a; Leng et al., 2016a; Hao et al., 2017) and malignant brain edema (Huang et al., 2019), which were common causes of ND after EVT. History of hypertension and lower ASPECTS were also observed to increase the risk of AD and SD. History of hypertension usually signifies a higher admission SBP (152 ± 22 vs. 139 ± 23 mmHg, *P* < 0.001 in our study) which only showed a moderate association with ND in this study (*P* = 0.069, Table 1). However, several studies have suggested that elevated admission SBP levels increase the risk of sICH (Mulder et al., 2017; Malhotra et al., 2020), ischemia progression (Goyal et al., 2017), early ischemic stroke recurrence (Leonardi-Bee et al., 2002) and cerebral edema (Leonardi-Bee et al., 2002), and consequently contribute to the occurrence of ND. A lower ASPECTS commonly indicates a higher NIHSS score with poor collaterals (Liebeskind et al., 2014a; Yoo et al., 2016), while both a higher NIHSS score and poor collaterals are considered major risk factors for ND.

There are limitations to this study. This was a retrospective single-center study with a limited sample size, especially for DD because of its low incidence. Further studies with a larger sample size in multiple centers are needed. Clinical confounding factors of ND are complicated, and not all of these factors were included. For example, post-stroke pneumonia, a common complication after stroke, was not included in this study because it is difficult to identify whether post-stroke pneumonia led to ND or ND resulted in post-stroke pneumonia in a logistic regression model. Finally, due to the unclear mechanism of ND and due to retrospective nature in this study, we failed to investigate the risk factors of ND according to its etiology, which needs to be explored in further studies.

Our study has clinical implications. ND at different time periods (AD, SD, and DD) predicted poor outcome, underlining the need to emphasize close neurological monitoring, especially within 24 h after EVT. The focus of monitoring should change as time progresses: history of hypertension, CE stroke, lower ASPECTS, and poor collaterals for AD (≤24 h); history of hypertension, ASPECTS, poor collaterals and unsuccessful recanalization for SD (24–72 h); and high NIHSS score for DD (>72 h). Recanalization rates should receive more attention during EVT. If recanalization is not achieved, prevention of hypovolemia (Arenillas et al., 2018), hypo- and hypertension (Biose et al., 2020; Raychev et al., 2020), hyperglycemia (Biose et al., 2020), and hyperuricemia (Menon et al., 2013) are primary targets for preserving collaterals. Other uncontrollable factors, including history of hypertension, CE stroke, lower ASPECTS and high NIHSS score, should also be considered in decision-making protocols before EVT. Families often expect patients to benefit more from EVT. We should prepare them with the fact that approximately 1/3 of patients experience ND, which implies a poor short-term prognosis.

Neurological deterioration by ≥4 NIHSS points occurring in one-third of patients with ischemic stroke undergoing EVT is a strong predictor for poor stroke outcomes. The risk factors for ND change as time progresses. Management of risk factors at different ND time periods might improve the prognosis of patients who undergo EVT in the future. Further large-scale studies are warranted to validate our findings and to delineate optimal criteria to prevent ND.

## Data Availability Statement

The raw data supporting the conclusions of this article will be made available by the authors, without undue reservation.

## Ethics Statement

The studies involving human participants were reviewed and approved by the Ethical Review Board of Yijishan Hospital in Wuhu, China (2019-039). The patients/participants provided their written informed consent to participate in this study.

## Author Contributions

ZL, HZ, and JH designed the study, analyzed all the data, and prepared the manuscript. ZZ and XH conceptualized the study, interpreted study data, and revised the manuscript. ZC and SZ performed the statistical analysis. QY collected clinical data and image data. All authors approved the final manuscript.

## Conflict of Interest

The authors declare that the research was conducted in the absence of any commercial or financial relationships that could be construed as a potential conflict of interest.
