# Supplementary material for: Time Course and Clinical Relevance of Neurological Deterioration After Endovascular Recanalization Therapy for Anterior Circulation Large Vessel Occlusion Stroke
Source: Front Aging Neurosci. 2021 Jun 29;13:651614. doi: 10.3389/fnagi.2021.651614 (PMC8277420; doi:10.3389/fnagi.2021.651614)
Supplement: Supplementary file 1 [file Table_1.docx]

Supplementary Material

**Supplementary Table 1** Baseline factors associated with stroke outcome at 90 days

| Variables | mRS≤ 2  N=148 | mRS> 2  N=195 | P value |
| --- | --- | --- | --- |
| Demographic characteristics | | | |
| Age, y, mean (SD) | 66.1 (10.7) | 70.4 (11.1) | < 0.001 |
| Female sex, n (%) | 54 (36.5) | 101 (51.8) | 0.005 |
| Past Medical History, n (%) | | | |
| Hypertension | 98 (66.2) | 145 (74.4) | 0.100 |
| Diabetes mellitus | 17 (11.5) | 40 (20.5) | 0.026 |
| Atrial fibrillation | 54 (36.5) | 118 (60.5) | < 0.001 |
| Antithrombotics | 28 (18.9) | 68 (34.9) | 0.001 |
| Clinical data | | | |
| Admission SBP, mean, (SD) | 145 (22) | 151 (23) | 0.012 |
| Admission DBP, mean (SD) | 81 (14) | 83 (14) | 0.231 |
| Admission NIHSS, median, (IQR) | 14 (11-17) | 17 (14-20) | < 0.001 |
| IV-tPA, n (%) | 17 (11.5) | 23 (11.8) | 0.930 |
| Occlusion site, n (%) |  |  | < 0.001 |
| ICA | 45 (30.4) | 103 (52.8) |  |
| MCA-M1 | 89 (60.1) | 77 (39.5) |  |
| MCA-M2, ACA | 14 (9.5) | 15 (7.7) |  |
| TOAST type, n (%) |  |  | < 0.001 |
| CE | 64 (43.2) | 134 (68.7) |  |
| LAA | 66 (44.6) | 43 (22.1) |  |
| Others | 18 (12.2) | 18 (9.2) |  |
| Radiological findings and procedural aspects | | | |
| ASPECTS, median (IQR) | 9 (8-10) | 8 (7-9) | < 0.001 |
| OTP, median (IQR) | 270 (224-325) | 260 (210-315) | 0.442 |
| PT, median (IQR) | 55 (40-80) | 73 (50-105) | < 0.001 |
| Good collaterals, n (%) | 93 (62.8) | 47 (24.1) | < 0.001 |
| Procedural modes, n (%) |  |  | 0.011 |
| Solitaire FR first | 108 (73.0) | 138 (70.8) |  |
| Inspiration first | 20 (13.5) | 45 (23.1) |  |
| Others | 20 (13.5) | 12 (6.2) |  |
| Remedial measures, n (%) | 16 (10.8) | 33 (16.9) | 0.109 |
| mTICI (2b/3), n (%) | 130 (87.8) | 120 (61.5) | < 0.001 |

mRS, modified Rankin Scale; SBP, systolic blood pressure; DBP, diastolic blood pressure; NIHSS, National Institutes of Health Stroke Scale; SD, standard deviation; IQR, interquartile range; IV‑rtPA, intravenous recombinant tissue plasminogen activator; ICA, internal carotid artery; MCA, middle cerebral artery; TOAST, Trial of Org 10172 in acute stroke treatment; CE, cardioembolic; LAA, large artery atherosclerosis; ASPECTS, the Alberta Stroke Program Early Computed Tomography Score; OTP, onset to puncture time; PT, procedural time; mTICI, modified Thrombolysis in Cerebral Infarction
